# Dynamic tuberculosis infection pattern: high conversion and reversion among persons with type 2 diabetes in India

**DOI:** 10.5588/pha.26.0039

**Published:** 2026-08-24

**Authors:** L. Devaraj, S. Subramanian, G. Ramaswamy, J.P. Tripathy, K. Soundappan, S.P. Babu, A.N. Aggarwal, K. Selvaraj, V. Venugopal, N. Agarwal, S. Sampath, J.E. John, A. Bhoyar, S. Sethi, N. Sachdeva, S. Sarkar, M. Rajaram, P.B. Narasimhan, M. Vijayageetha, D. Nair, P. Thekkur, P. Chinnakali

**Affiliations:** 1 Jawaharlal Institute of Postgraduate Medical Education and Research, Puducherry, India;; 2 All India Institute of Medical Sciences, Bibinagar, India;; 3 All India Institute of Medical Sciences, Nagpur, India;; 4 Tufts University of School of Medicine, Boston, MA, USA;; 5 Postgraduate Institute of Medical Education and Research, Chandigarh, India;; 6 Dahdaleh Institute for Global Health Research, School of Global Health, York University, Toronto, ON, Canada;; 7 All India Institute of Medical Sciences, Madurai, India;; 8 All India Institute of Medical Sciences, Deoghar, India;; 9 Independent researcher, Scottsdale, AZ, USA;; 10 Centre for Operational Research, International Union Against Tuberculosis and Lung Disease, Paris, France;; 11 UNICEF, UNDP, World Bank, WHO Special Programme for Research and Training in Tropical Diseases (TDR), Geneva, Switzerland.

**Keywords:** tuberculosis, interferon-gamma release assay, latent TB, TB prevention, SORT-IT, operational research

## Abstract

**SETTINGS:**

A multicentric study in eight primary health centres across four geographically diverse regions of India (Puducherry, Telangana, Maharashtra, and Chandigarh).

**OBJECTIVES:**

Among persons with diabetes (PWD) registered for care, to determine the conversion and reversion rates of tuberculosis infection (TBI) at 6 and 12 months and its associated factors.

**DESIGN:**

Cohort study with serial interferon-gamma release assay (IGRA) at baseline and at 6 and 12 months after excluding active TB. Conversion (baseline TBI negative to positive) and reversion (baseline TBI positive to negative) was assessed, and modified Poisson regression was used to identify associated factors.

**RESULTS:**

Of 750 recruited, 748 PWD underwent baseline IGRA (excluding two newly diagnosed TB), and 339 (45%) were positive. At 12 months, reversion among baseline positives was 38% (95% confidence interval [CI]: 32–44), while conversion among baseline negatives was 22% (95% CI: 17–26). Tobacco non-use was associated with reversion, while older age (≥60 years) and lower body mass index compared to obesity (>25 kg/m^2^) were associated with conversion.

**CONCLUSION:**

High rates of IGRA conversion and reversion among PWD highlight the dynamic nature of TBI and question the utility of single time-point TBI testing for making decisions regarding initiation of TB preventive therapy.

## Graphical abstract

**Figure:**
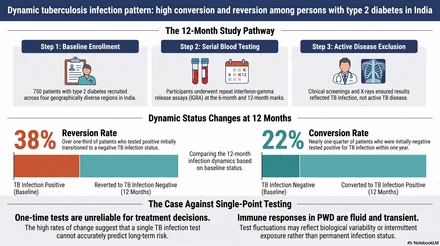

The WHO defines tuberculosis infection (TBI) as a persistent immune response to *Mycobacterium tuberculosis* antigens without clinically active TB. Approximately 5%–10% of individuals with TBI may progress to active TB disease during their lifetime.^1^ Since many TB cases arise from reactivation of TBI, targeting high-risk groups for testing and preventive therapy helps shrink the reservoir of infection. The common diagnostic tools for TBI are the tuberculin skin test (TST) and interferon-gamma release assay (IGRA),^2^ with IGRA preferred due to higher specificity in bacille Calmette-Guérin (BCG)-vaccinated populations and the need for a single visit, despite higher cost and laboratory requirements.

TBI represents a dynamic immunological state, which has significant implications for how these diagnostic tools are interpreted and used programmatically. TBI status can change over time, leading to conversion (TBI negative to TBI positive) or reversion (TBI positive to TBI negative) due to various factors like test variability, variability in exposure dynamics, and transient immune responses.^3^ The annual conversion and reversion rates using IGRA range between 2%–6% and 10%–20%, respectively, among high-risk populations such as health care workers (HCWs) and immune-compromised individuals.^4,5^ Another study among HCWs in India reported a baseline TBI prevalence of 37.7%, with conversion and reversion rates of 5.6 and 11.7 per 100 person-years, respectively.^6^ Besides these groups, other high-risk groups must be identified for TBI screening, and persons with diabetes (PWD) are one such group.^7^ Diabetes mellitus (DM) increases the risk of progression from infection to active TB disease and also makes an individual more vulnerable to TBI through various cellular and molecular mechanisms.^8–10^ A recent study from India reported that nearly half of the type 2 DM (T2DM) patients had TBI.^11^ Despite this evidence, the National Tuberculosis Elimination Programme (NTEP) of India does not routinely recommend TBI screening and TB preventive treatment (TPT) for PWD with TBI.^12^ This is partly due to limited understanding of TBI dynamics in this group, particularly in the context of impaired immunity. Additionally, PWD are not currently prioritised for TPT, and systematic screening for TB and TBI is not well integrated with non-communicable disease (NCD) programmes.^3^ As a result, uncertainty remains regarding infection acquisition, clearance, and stability over time in PWD. Addressing these gaps is essential for informing programmatic decisions regarding whom to screen, the optimal frequency of screening, and which individuals would benefit most from TPT.

Therefore, we conducted this multicentric investigation among adult PWD (aged >18 years) registered for care in selected primary health centres (PHCs) across four geographically diverse jurisdictions of India to determine the conversion and reversion rates at 6 and 12 months since recruitment and its associated socio-demographic, behavioural, and clinical characteristics.

## METHODS

### General setting

India accounts for nearly 27% of the global TB burden, with an estimated 2.61 million individuals diagnosed with TB in 2024.^13^ It is also the diabetes capital of the world, with over 90 million PWD.^14^ The NTEP recommends TBI screening and TPT for selected vulnerable groups, particularly people living with HIV, household contacts of pulmonary TB patients, and immunocompromised individuals.^15^ Currently, routine TBI screening for PWD is not practised under NTEP and there are no guidelines on providing TPT in PWD.^12^

### Specific setting

This study was conducted in eight PHCs across four regions of India: Puducherry, Telangana, Maharashtra, and Chandigarh between March 2023 and September 2025. These PHCs provide basic diabetes management services including blood glucose monitoring, lifestyle counselling, and medication refills through their NCD clinics. The details of care and follow-up visits of these registered patients are maintained in an NCD register.

### Study population

PWD (only T2DM) aged ≥18 years registered for care at selected urban and rural PHCs in 2023 across the four study sites were included. PWD with active TB disease and pregnancy were excluded.

### Sample size and sampling

The sample size was calculated using OpenEpi (v3.01) for a larger multicentric study, assuming a 33% TBI prevalence among PWD, 20% relative precision, and 5% alpha error. The estimated sample size was 196 per site, increased to 216 after accounting for 10% loss to follow-up (total 864 across four sites). One urban and one rural PHC were selected from the field practice area of the participating tertiary care teaching institutions. Around 100 PWD each from the urban and rural PHCs were chosen by simple random sampling from the line list of PWD maintained at these centres.

### Data collection

A structured questionnaire was used to collect information on socio-demographic (age, gender, education, socio-economic status, residence), behavioural (body mass index (BMI), alcohol, and tobacco use in the past 1 year), and clinical characteristics (TB symptoms, duration of DM, glycaemic control). Anthropometric measurements (height and weight) were recorded, and BMI was classified as per Asian criteria.^16^ Approximately 6 mL of venous blood was collected by trained laboratory technicians in labelled tubes and transported in cold boxes to the respective institution’s laboratory on the same day for IGRA and glycosylated haemoglobin (HbA1c) estimation. PWD with symptoms suggestive of TB underwent chest X-ray and sputum examination as per programmatic guidelines to rule out active TB, following which IGRA testing was performed. TBI testing was performed using fourth-generation QuantiFERON^®^-TB Gold Plus (QFT-Plus) kits (QIAGEN, Hilden, Germany),^17^ while HbA1c was assessed using high-performance liquid chromatography.^18^ PWD without symptoms suggestive of TB but with a positive IGRA result underwent further evaluation, including chest radiography, to exclude active TB. A diagnosis of TBI was confirmed only after active TB was ruled out by a chest physician. Follow-up assessments were conducted at 6 and 12 months, during which IGRA and HbA1c testing were repeated for all participants, along with assessment for symptoms suggestive of TB. PWD diagnosed with active TB at any stage were excluded from the analysis.

### Operational definitions

TBI positive was defined as an interferon-γ (IFN-γ) response in either TB1 or TB2 antigen tube minus the Nil control ≥0.35 IU/mL and at least 25% of the Nil value. TBI negative was defined as a response <0.35 IU/mL, or ≥0.35 IU/mL but <25% of the Nil value, provided the Mitogen control minus Nil was ≥0.5 IU/mL, indicating an adequate immune response. Results were classified as indeterminate if the Nil value was >8.0 IU/mL or if the Mitogen minus Nil was <0.5 IU/mL, suggesting high background levels or inadequate immune response. All values were expressed in IU/mL, with Nil values expected to be <8.0 IU/mL for valid interpretation.^19^

#### PWD

PWD were defined as those with an HbA1c >6.5% or known case of DM (either documentation of blood sugar tests or prescription of medications for diabetes).

For reversion and conversion, we used two definitions (most stringent and least stringent criteria) to assess whether variations in definitions produce different rates of reversion and conversion events.

#### Reversion

Among those who were positive for TBI (≥0.35 IU/mL) at the baseline, we used two definitions for reversion. One was based on the IGRA cut-off values of <0.20 IU/mL in TB1 and TB2 tubes (most stringent criteria) and the other was based on cut-off values of <0.35 IU/mL in TB1 and TB2 tubes (least stringent criteria).^5^

#### Conversion

Among those who were negative (<0.35 IU/mL) at the baseline, we used two definitions for conversion. One was based on the IGRA cut-off values of >0.70 IU/mL in TB1 or TB2 tubes (twice the manufacturer-recommended diagnostic cut-off and the most stringent criteria)^4^ and the other was based on cut-off values of ≥0.35 IU/mL in TB1 or TB2 tubes (as recommended by the Centers for Disease Control and Prevention and the least stringent criteria).^20^

### Data analysis

Data were exported from EpiCollect5 (V87.2.1, 2025) and analysed in Stata/MP version 18.5 (StataCorp LLC). Baseline TBI at recruitment and conversion and reversion of TBI at 6 and 12 months are reported as proportion with 95% confidence intervals (CIs). Change in TBI status from baseline at 6 and 12 months is depicted using a flow diagram. We considered the baseline TBI status for estimation of conversion and reversion. Unadjusted and adjusted modified Poisson regression analysis with robust variance estimation was used to explore PWD characteristics associated with reversion at the end of 12 months and effect sizes are presented as adjusted relative risks (aRR) with 95% CIs. Due to low number of events, and to avoid unstable estimates, multivariable analysis for conversion was not performed.

### Ethical statement

Ethics approval was obtained from JIPMER Institutional Ethics Committee (JIP/IEC/2022/079) and all four participating institutions. Written informed consent was obtained from all participants prior to recruitment.

## RESULTS

A total of 750 PWD were recruited across four study sites. Most belonged to the age group >60 years (346, 46.1%). Nearly half were female (408, 54.4%) and were from urban areas (378, 50.4%). One fifth reported alcohol use in the past 1 year (150, 20%) and 13.5% (n = 101) reported tobacco use in the last year. Nearly half had DM for more than 5 years (364, 48.5%) and 39.9% (n = 299) had glycaemic control at baseline (Table 1).

**TABLE 1.** Socio-demographic, behavioural, and clinical characteristics of persons with type 2 diabetes mellitus (DM) tested for tuberculosis (TB) infection registered for care in selected primary health centres in four jurisdictions of India during 2023–2025 (N = 750).

Characteristic	Category	n (%)
Total		750 (100)
Age group, years	18–45	111 (14.8)
	46–59	293 (39.1)
	>60	346 (46.1)
Sex	Male	342 (45.6)
	Female	408 (54.4)
Residence	Urban	378 (50.4)
	Rural	372 (49.6)
Education status	No formal education	316 (42.1)
	Up to Primary	127 (16.9)
	Up to Secondary	101 (13.5)
	High school and above	206 (27.5)
Socio-economic status^A^ (per capita income in INR/month)	Class I (≥8,822)	41 (5.5)
	Class II (4,411–8,821)	123 (16.4)
	Class III (2,647–4,410)	273 (36.4)
	Class IV (1,323–2,646)	221 (29.5)
	Class V (<1,223)	92 (12.2)
Tobacco use in the past 1 year	Yes	101 (13.5)
	No	649 (86.5)
Alcohol use in the past 1 year	Yes	150 (20.0)
	No	600 (80.0)
Household history of TB	Yes	29 (3.9)
	No	721 (96.1)
Body mass index, kg/m^2^	Underweight (<18.5)	32 (4.3)
	Normal (18.5–22.9)	177 (23.6)
	Overweight (23–24.9)	130 (17.3)
	Obese (>25)	411 (54.8)
Duration of DM, years	<5	386 (51.5)
	≥5	364 (48.5)
Glycaemic status^B^	Controlled (HbA1c ≤ 7)	299 (39.9)
	Uncontrolled (HbA1c > 7)	451 (60.1)

A As per Modified BG Prasad Classification for 2023.

B Glycaemic status reference values were adopted from the National Programme for Prevention and Control of Non-Communicable Diseases standard treatment protocol for type 2 DM.

### Serial IGRA testing

Figure 1 presents the results of IGRA serial testing at baseline and at 6 and 12 months after recruitment. At baseline, 748 were tested for IGRA after excluding two cases of active TB. At 6 months, 625 were tested (excluding 121 participants lost to follow-up and two cases of active TB), and 593 were tested at 12 months (excluding 147 lost to follow-up and two cases of active TB).

**FIGURE 1.**
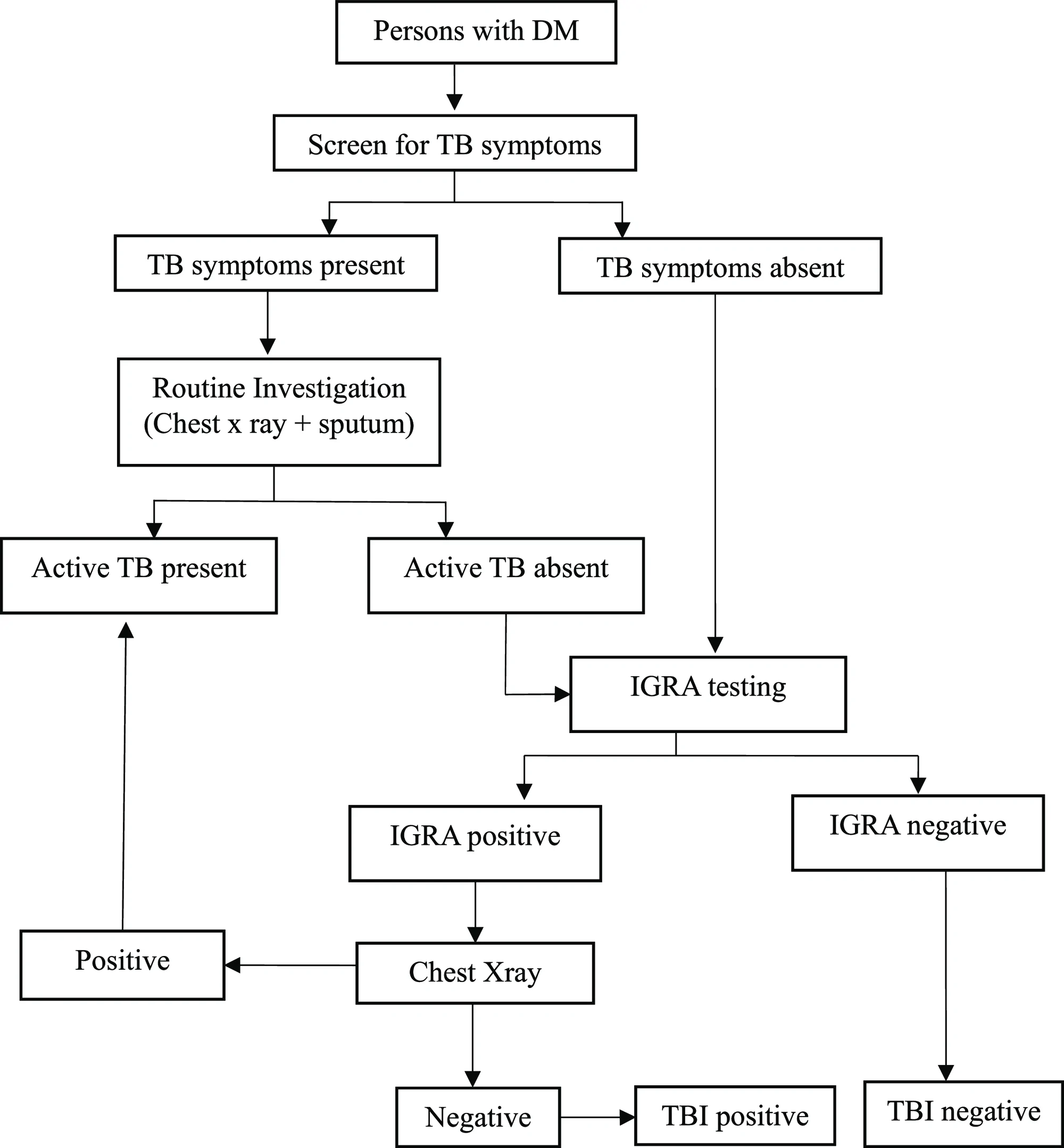
Diagnostic algorithm followed in this study for detecting TB infection (TBI) in persons with diabetes mellitus (DM) registered in selected primary health centres in four jurisdictions of India during 2023–2025. TB = tuberculosis; IGRA = interferon-gamma release assay.

### Persistence of TBI status

Of 748 tested at baseline, 339 (45.3%) PWD were TBI positive. At 6 months, 209/337 (62.0%) remained TBI positive, of whom 150/337 (44.5%) remained persistently TBI positive at 12 months. Of 409 TBI negative at baseline, 286 (69.9%) remained negative at 6 months, and 218/408 (53.4%) continued to remain TBI negative at 12 months, indicating stable TBI-negative status over time (Figure 2).

**FIGURE 2.**
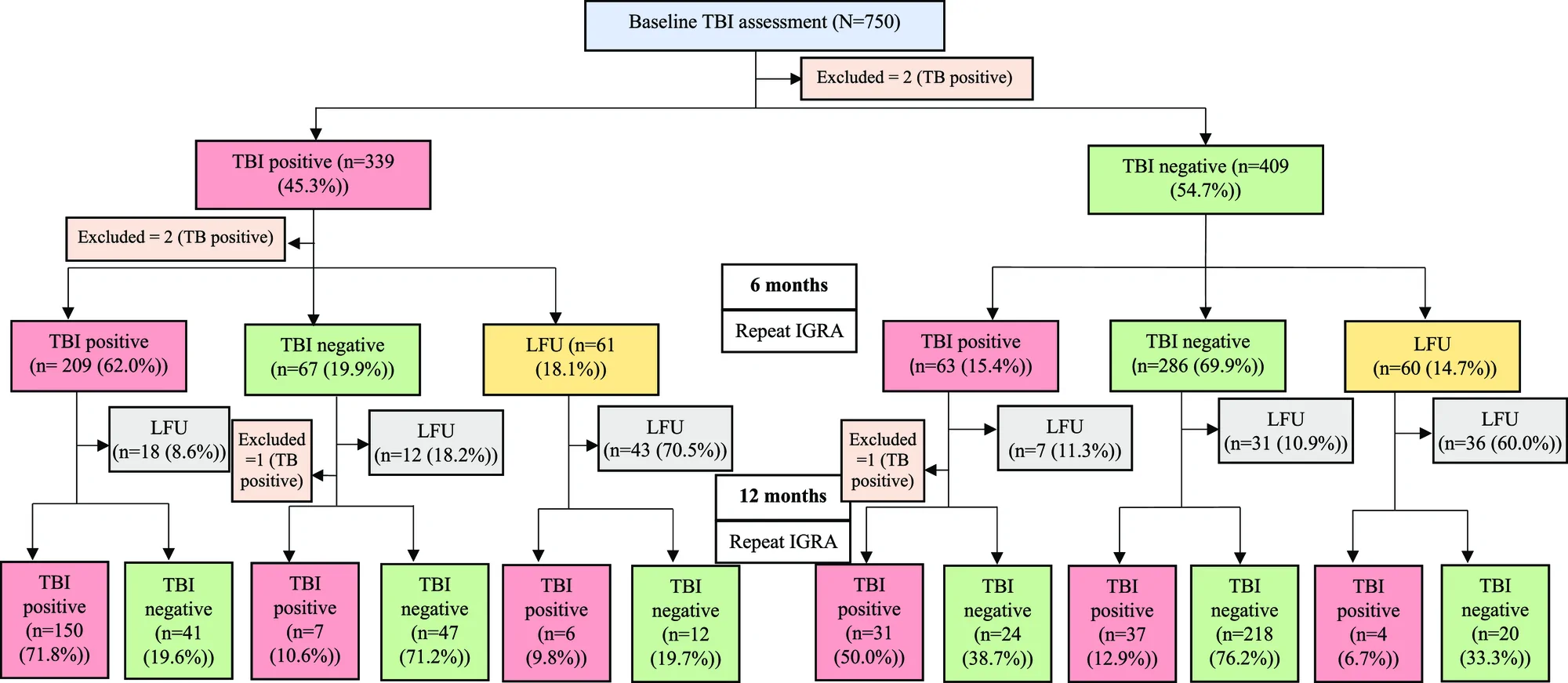
Results of IGRA serial testing at 6 and 12 months after recruitment among persons with diabetes mellitus (DM) in four jurisdictions of India during 2023–2025. TB = tuberculosis; TBI = TB infection; LFU = lost to follow-up; IGRA = interferon-gamma release assay; Reversion: Participants who were positive (≥0.35 IU/mL) for TBI and then turned negative during the subsequent follow-up with IGRA cut-off values of <0.20 IU/mL in TB1 and TB2 tubes. Conversion: Participants who were negative (<0.35 IU/mL) for TBI who turned positive during the subsequent follow-up with IGRA cut-off values of >0.70 IU/mL in TB1 or TB2 tubes.

### Reversion and conversion rates

Of the 263 participants who were TBI positive at baseline and followed up for 12 months, 100 reverted to TBI negative (38%, 95% CI: 32.1–44.2) at 12 months. Tobacco non-use in the last 1 year (aRR: 2.52, 95% CI: 1.31–4.85, *P* < 0.01) was significantly associated with reversion at 12 months (Table 2). Among 334 participants who were TBI negative at baseline and followed up until 12 months, the incidence of conversion at 12 months was 21.6% (95% CI: 17.3–26.4). Age 60 years and above and lower BMI were significantly associated with conversion at 12 months. Glycaemic control was not found to be associated with reversion and conversion. At 6 months, incidences of reversion and conversion were 19.9% (95% CI: 15.8–24.5) and 15.4% (95% CI: 12.0–19.3), respectively (Table 3). The results of IGRA serial testing using the alternative least stringent criteria for defining reversion and conversion are available at: https://doi.org/10.6084/m9.figshare.31883521. The conversion and reversion rates at 12 months using the alternative least stringent criteria are 27% and 40%, respectively.

**TABLE 2.** Association of socio-demographic, behavioural, and clinical characteristics with reversion of TB infection at 12 months among persons with type 2 diabetes mellitus who were positive at baseline in four jurisdictions in India during 2023–2025 (N = 263).

Characteristic	Total	Reversion,^A^ n (%)	cRR (95% CI)	aRR (95% CI)	*P* value
Total	263	100 (38.0)			
Age group, years					
18–45	37	17 (45.9)	1.35 (0.88–2.07)	1.28 (0.83–1.95)	0.25
46–59	99	40 (40.4)	1.19 (0.84–1.67)	1.04 (0.75–1.47)	0.78
≥60	127	43 (33.9)	1	1	–
Gender					
Men	131	50 (38.2)	1.01 (0.74–1.37)	1.01 (0.63–1.44)	0.99
Women	132	50 (37.9)	1	1	–
Residence					
Urban	130	48 (36.9)	1	1	–
Rural	133	52 (39.1)	1.05 (0.77–1.44)	1.02 (0.76–1.39)	0.86
Socio-economic status^B^ (per capita income in INR/month)					
Classes I and II	46	13 (28.3)	1	1	–
Class III	101	42 (41.6)	1.47 (0.87–2.46)	1.36 (0.84–2.22)	0.21
Classes IV and V	116	45 (38.8)	1.37 (0.82–2.29)	1.28 (0.78–2.10)	0.32
Tobacco use in the past 1 year^C^					
Yes	51	9 (17.7)	1	1	–
No	212	91 (42.9)	2.43 (1.31–4.48)**	2.52 (1.31–4.85)**	**<0.01**
Alcohol use in the past 1 year^C^					
Yes	55	26 (47.3)	1.32 (0.95–1.85)	1.45 (1.00–2.13)	0.05
No	208	74 (35.6)	1	1	–
Household history of TB					
Yes	10	3 (30.0)	1	1	
No	253	97 (38.3)	1.27 (0.48–3.33)	1.25 (0.49–3.19)	0.63
Body mass index, kg/m^2^					
Underweight (<18.5)	7	3 (42.9)	1.16 (0.48–2.81)	1.21 (0.49–2.95)	0.67
Normal (18.5–22.9)	61	24 (39.3)	1.06 (0.73–1.56)	1.11 (0.76–1.62)	0.58
Overweight (23.0–24.9)	51	20 (39.2)	1.06 (0.72–1.59)	1.13 (0.76–1.68)	0.53
Obese (>25)	144	53 (36.8)	1	1	–
Glycaemic status^D^					
Controlled (HbA1c ≤ 7)	103	40 (38.8)	1.03 (0.75–1.41)	1.02 (0.74–1.40)	0.89
Uncontrolled (HbA1c > 7)	160	60 (37.5)	1	1	–

Bold values are statistically significant (*P* < 0.05)

*P < 0.05; **P < 0.01.

TB = tuberculosis; CI = confidence interval; cRR = crude relative risk; aRR = adjusted relative risk; IGRA = interferon-gamma release assay.

A Reversion: ≥0.35 IU/ml at baseline and follow-up IGRA values of <0.20 IU/ml in TB1 and TB2 tubes.

B As per Modified BG Prasad Classification for 2023.

C Information collected at Baseline.

D Glycaemic status reference values were adopted from the National Programme for Prevention and Control of Non-Communicable Diseases standard treatment protocol for type 2 diabetes mellitus.

**TABLE 3.** Association of socio-demographic, behavioural, and clinical characteristics with conversion of TB infection at 12 months among those who were negative at baseline in four jurisdictions of India during 2023–2025 (N = 334).

Characteristic	Total	Conversion,^A^ n (%)	cRR (95% CI)	*P* value
Total	334	72 (21.6)		
Age group, years				
18–45	62	12 (19.3)	1.18 (0.62–2.25)	0.59
46–59	129	17 (16.3)	1	1
≥60	143	30 (27.3)	1.67 (1.04–2.69)	**0.03**
Gender				
Men	152	31 (20.4)	1	–
Women	182	41 (22.5)	1.10 (0.73–1.67)	0.64
Residence				
Urban	157	36 (22.9)	1.12 (0.74–1.69)	0.56
Rural	177	36 (20.3)	1	–
Socio-economic status^B^ (per capita income in INR/month)				
Classes I and II	85	19 (22.4)	1.12 (0.65–1.93)	0.66
Class III	116	23 (19.8)	1	–
Classes IV and V	133	30 (22.6)	1.13 (0.70–1.84)	0.60
Tobacco use in the past 1 year^C^				
Yes	39	4 (10.3)	1	–
No	295	68 (23.1)	2.25 (0.86–5.81)	0.09
Alcohol use in the past 1 year^C^				
Yes	58	8 (13.8)	1	–
No	276	64 (23.2)	1.68 (0.85–3.31)	0.13
Household history of TB				
Yes	6	2 (33.3)	1.56 (0.49–4.93)	0.45
No	328	70 (21.3)	1	–
Body mass index, kg/m^2^				
Underweight (<18.5)	16	5 (31.3)	2.08 (0.93–4.65)	0.07
Normal (18.5–22.9)	78	20 (25.6)	1.71 (1.02–2.85)	**0.03**
Overweight (23–24.9)	53	19 (35.9)	2.39 (1.46–3.93)	**<0.01**
Obese (>25)	187	28 (14.9)	1	–
Glycaemic status^D^				
Controlled (HbA1c ≤ 7)	136	31 (22.8)	1.10 (0.72–1.66)	0.64
Uncontrolled (HbA1c > 7)	198	41 (20.7)	1	–

Bold values are statistically significant (*P* < 0.05)

*P < 0.05; **P < 0.01.

TB = tuberculosis; CI = confidence interval; cRR = crude relative risk; IGRA = interferon-gamma release assay.

A Conversion: <0.35 IU/mL at baseline and cut-off IGRA values of >0.70 IU/mL in TB1 or TB2 tubes.

B As per Modified BG Prasad Classification for 2023.

C Information collected at Baseline.

D Glycaemic status reference values were adopted from the National Programme for Prevention and Control of Non-Communicable Diseases standard treatment protocol for type 2 diabetes mellitus.

## DISCUSSION

This is the first study from India reporting the results of serial IGRA testing in a large multicentric cohort of PWD. The study demonstrated the dynamic nature of TBI in this population, with substantial annual conversion (22%) and reversion rates (38%), highlighting both the instability of IGRA results over time and the complexity of interpreting TBI status among PWD.

A key finding in our study is the relatively high conversion rate at 12 months, which is higher than many reports from other populations.^4,21,22^ IGRA conversion may not always reflect incident infection but could arise from biological variability, intermittent antigen exposure, or variability around the diagnostic threshold.^22^ Studies in immunocompromised populations, such as those receiving biologics or living with HIV, have reported variable conversion rates ranging from 9% to 30%.^23,24^ This suggests that in PWD, who have altered immune responses, conversion may reflect a combination of true incident infection and immune response variability. We believe that because IGRA results are influenced by biological and test-related variability, instead of reporting IGRA tests as dichotomous values, the actual IFN-γ level or rising levels may be more useful in identifying subgroups at risk of developing active TB.^22^ Pai and O’Brien^3^ referred to ‘strong conversion’ which may be different from that of a ‘weak conversion’ based on high or rising levels of IFN-γ, rather than a dichotomous cut-off. Andersen and colleagues^25^ also hypothesised that high and/or rising levels of IFN-γ may predict development of overt clinical disease in the near future, which needs confirmation in future studies.

The high reversion rate found in this study is consistent with prior literature reporting reversion ranging from 35% to 44%.^21,26^ A systematic review of IGRA serial testing among HCWs also reported reversion ranging between 22% and 71%.^22^ This finding has implications for timing and repeat testing of TBI screening in this population and TPT initiation. Reversions could mean clearing of TBI, biological variations among IGRA positives, or variability in laboratory and test procedures.^27^ Hill et al.^21^ suggested that IGRA responses are inherently transient and may require continued exposure to TB antigens to maintain positivity. IGRA reversions are more likely when the baseline test results are discordant (i.e., positive by IGRA but negative by TST) or weakly positive (just above the diagnostic threshold).^3^ This again emphasises the need for using changes in quantitative IGRA values rather than a dichotomous status based on set criteria.

The present study has important policy implications. Serial IGRA testing in PWD may better capture the dynamic nature of TBI than a single time-point assessment, but its programmatic value remains uncertain because repeated testing increases costs and requires laboratory infrastructure. Although the recently introduced Cy-TB skin test under NTEP offers a more practical alternative for programmatic TBI screening than IGRA, it also requires a return visit at 48 h for test reading, which may affect feasibility in routine services. Therefore, before adopting serial TBI testing strategies for diabetes patients in routine care, studies should define biologically meaningful thresholds for conversion and reversion, compare IGRA with Cy-TB in terms of accuracy and operational feasibility, and evaluate whether either strategy improves identification of those most likely to benefit from TPT.

This study addressed an important evidence gap by providing longitudinal data on TBI dynamics among PWD, with serial IGRA testing at multiple time points allowing estimation of both conversion and reversion rates. Inclusion of multiple geographically diverse sites across India enhances generalisability. Additionally, standardised laboratory methods (QFT-Plus and HbA1c for glycaemic control) and systematic exclusion of active TB improve the validity of TBI classification. This study also has some limitations. Loss to follow-up (∼20%) may introduce attrition bias. Use of IGRA without TST limits comparative interpretation of test performance. High reversion rates may reflect biological variability or assay-related fluctuations, raising concerns about misclassification. The study may be underpowered to detect factors associated with conversion due to fewer number of event counts. Additionally, detailed data on TB exposure and environmental risk factors were lacking; therefore, infection dynamics could not be fully assessed.

## CONCLUSION

High conversion and reversion rates indicate that TBI status among PWD is dynamic over time. These findings suggest that a single IGRA result may not be sufficient for programmatic decision making, and further evidence is needed to determine the role of repeat testing in routine TB prevention services.
